# Exploring Phosphatidylethanol Cutoffs for Self‐Reported Unhealthy Alcohol Use: An International Multi‐Site Analysis

**DOI:** 10.1111/acer.70361

**Published:** 2026-06-25

**Authors:** Pamela M. Murnane, Fan Xia, Majid Afshar, Jennifer L. Brown, Gabriel Chamie, Robert L. Cook, Marie Claude Couture, Ralph J. DiClemente, Robin Fatch, Tekeda Ferguson, Joel M. Francis, Jessica E. Haberer, Karen R. Jacobson, Amy C. Justice, Saidi Kapiga, Theresa W. Kim, Evgeny Krupitsky, Gregory M. Marcus, Patricia E. Molina, Winnie R. Muyindike, Bronwyn Myers, Kimberly Page, Shane A. Phillips, Mariann R. Piano, Veronica L. Richards, Kaku So‐Armah, Scott Stewart, Mark S. Sulkowski, Phyllis C. Tien, Sarah Woolf‐King, Judith A. Hahn

**Affiliations:** ^1^ Department of Epidemiology and Biostatistics and the Institute for Global Health Sciences University of California, San Francisco San Francisco California USA; ^2^ Department of Epidemiology and Biostatistics University of California, San Francisco San Francisco California USA; ^3^ Department of Medicine, School of Medicine and Public Health University of Wisconsin–Madison Madison Wisconsin USA; ^4^ Department of Psychological Sciences Purdue University West Lafayette Indiana USA; ^5^ Department of Medicine University of California, San Francisco San Francisco California USA; ^6^ Department of Epidemiology University of Florida Gainesville Florida USA; ^7^ University of San Francisco San Francisco California USA; ^8^ Department of Social and Behavioral Sciences NYU School of Global Public Health New York New York USA; ^9^ Comprehensive Alcohol‐HIV/AIDS Research Center Louisiana State University Health Sciences Center New Orleans Louisiana USA; ^10^ Epidemiology Program, School of Public Health Louisiana State University Health Sciences Center New Orleans Louisiana USA; ^11^ Department of Family Medicine and Primary Care, School of Clinical Medicine, Faculty of Health Sciences University of the Witwatersrand Johannesburg South Africa; ^12^ Massachusetts General Hospital, Center for Global Health Boston Massachusetts USA; ^13^ Department of Medicine Boston University Chobanian and Avedisian School of Medicine Boston Massachusetts USA; ^14^ United States Department of Veterans Affairs VA Connecticut Healthcare System West Haven Connecticut USA; ^15^ Schools of Medicine and Public Health Yale University New Haven Connecticut USA; ^16^ Mwanza Intervention Trials Unit Mwanza Tanzania; ^17^ Department of Infectious Disease Epidemiology and International Health London School of Hygiene & Tropical Medicine London UK; ^18^ First Pavlov State Medical University St. Petersburg Russia; ^19^ V.M. Bekhterev National Medical Research Center for Psychiatry and Neurology St. Petersburg Russia; ^20^ Department of Physiology, School of Medicine Louisiana State University Health Sciences Center New Orleans Louisiana USA; ^21^ Department of Internal Medicine Mbarara University of Science and Technology Mbarara Uganda; ^22^ Curtin enAble Institute, Faculty of Health Sciences Curtin University Bentley Western Australia Australia; ^23^ Mental Health, Alcohol, Substance Use and Tobacco Research Unit South African Medical Research Council Tygerberg South Africa; ^24^ Department of Psychiatry and Mental Health University of Cape Town Cape Town South Africa; ^25^ Department of Internal Medicine University of New Mexico Albuquerque New Mexico USA; ^26^ Department of Physical Therapy University of Illinois at Chicago Chicago Illinois USA; ^27^ Center for Research Development and Scholarship Vanderbilt University Nashville Tennessee USA; ^28^ TSET Health Promotion Research Center University of Oklahoma Health Sciences Tulsa Oklahoma USA; ^29^ Department of Family Medicine, Division of Addiction Medicine University at Buffalo Buffalo New York USA; ^30^ School of Medicine Johns Hopkins University Baltimore Maryland USA; ^31^ San Francisco VA Health Care System San Francisco California USA; ^32^ Department of Psychology Syracuse University Syracuse New York USA; ^33^ Departments of Medicine and Epidemiology and Biostatistics University of California, San Francisco San Francisco California USA

**Keywords:** alcohol drinking, biomarkers, cutoffs, metabolism, phosphatidylethanol, self‐report

## Abstract

**Background:**

Unhealthy alcohol use is a preventable cause of morbidity and mortality, yet screening is hampered by inaccurate reporting. Phosphatidylethanol (PEth) is a biomarker that quantifies total drinking over the past 2–4 weeks, but PEth cutoffs for unhealthy drinking have not been well‐examined.

**Methods:**

We pooled data from 22 studies (11,088 persons globally) that previously collected PEth and self‐reported alcohol use. Within a 90% training set, we calculated PEth cutoffs per Youden's *J* in 1000 bootstrapped samples and explored differences by region, age, sex, race/ethnicity, body mass index (BMI), HIV status, hemoglobin level, and an indirect serum marker of liver fibrosis, FIB‐4. For each cutoff, we estimated sensitivity, specificity, and positive and negative predictive values in a 10% validation dataset. We used two definitions for self‐reported unhealthy drinking per Alcohol Use Disorders Identification Test Consumption (AUDIT‐C) and National Institute on Alcohol Abuse and Alcoholism (NIAAA).

**Results:**

Optimal PEth cutoffs using self‐reported alcohol use as the reference standard differed substantially by region. The cutoff for AUDIT‐C‐measured unhealthy alcohol use in studies from the United States (US) was 14.0 ng/mL (95% CI: 12.3–18.6) with 73.0% sensitivity (95% CI: 67.3–79.1) and 77.4% specificity (95% CI: 73.2–81.4); and was 65.7 ng/mL (95% CI 19.3–90.7) in studies from Africa, with 71.7% sensitivity (95% CI: 64.4–78.7) and 65.2% specificity (95% CI: 58.1–72.7). Cutoffs for AUDIT‐C did not differ between subgroups in the US, but within Africa, cutoffs were higher for men and lower for those with BMI ≥ 25 kg/m^2^. Cutoffs for NIAAA defined unhealthy alcohol use were similar to those using the AUDIT‐C.

**Conclusions:**

Using self‐report as the reference standard, PEth cutoffs differed substantially by region and by some other characteristics, which may be attributable to differences in PEth formation, elimination and/or reporting bias. Further work using objective gold‐standard measures of alcohol consumption is needed for more definitive conclusions.

## Graphical abstract

Phosphatidylethanol (PEth) is a biomarker that quantifies total drinking over the past 2–4 weeks, but PEth cutoffs for unhealthy drinking have not been well‐examined. Using self‐report as the reference standard, we estimated PEth cutoffs in a multinational sample and found substantial variability in cutoffs by region and by some other characteristics.

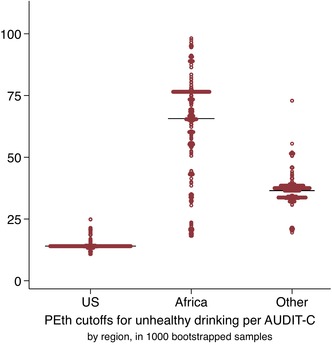

## 1 Introduction

Alcohol use is a leading cause of morbidity and mortality globally, with a clear dose–response relationship between levels of consumption and health risks (GBD 2016 Alcohol Collaborators 2018). Both prevention and treatment of unhealthy alcohol use, defined as drinking at or above a level that can have negative health consequences (Krist and Bradley 2025), alcohol use disorder, and alcohol‐associated morbidities depend on adequate screening and assessment of alcohol consumption to enable referral to appropriate evidence‐based interventions. However, timely and accurate assessment of alcohol intake is challenging for several reasons. For instance, care providers often lack the time, privacy, training, or skills to openly discuss alcohol use with patients (McNeely et al. 2018; Williams et al. 2015). Patients may misreport consumption due to fear of stigma from disclosure of unhealthy behavior (Davis et al. 2010), incomplete recall, or uncertainty regarding the quantity of drinks consumed given variability in sizes and alcohol content (Kerr and Stockwell 2012).

There is growing interest in the use of objective measures to more accurately assess alcohol use and provide a platform for counseling and referral. Phosphatidylethanol (PEth) is a direct ethanol metabolite that is formed only in the presence of ethanol through modification of plasma membrane phospholipids and accumulates in red blood cells. Thus, in contrast to other biomarkers, which are more rapidly eliminated from circulation, PEth is detectable in blood up to 4 weeks following heavy alcohol use, possibly longer for those with very high levels (de Bejczy et al. 2026; Luginbühl et al. 2019). Additionally, PEth is highly sensitive for detection of recent consumption (Helander et al. 2019; Tawiah et al. 2022; Torp et al. 2025). PEth concentrations are correlated with the quantity of alcohol intake (Aradottir et al. 2006), and PEth has been shown to outperform other biomarkers in detection of alcohol use (Afshar et al. 2022; Årving et al. 2021; Cameron et al. 2023; Helander et al. 2012; Neumann et al. 2020), making it a highly promising adjunct to self‐reported drinking to enhance screening and assessment. However, to facilitate interpretation, PEth concentration thresholds that align with clinically meaningful levels of alcohol consumption are needed. A general consensus has been reached that PEth concentrations ≤ 20 ng/mL reflect recent abstinence or very light use, and levels ≥ 200 ng/mL reflect chronic excessive use (Luginbuhl et al. 2022; Ulwelling and Smith 2018; Van Uytfanghe and Stove 2023). A wide range of thresholds have been proposed to discriminate unhealthy alcohol use from low use or abstinence (Perilli et al. 2023) including a recent large study of nearly 25,000 persons from Norway (Skråstad et al. 2023), yet no consensus has been reached to date.

Identifying a threshold for unhealthy alcohol use is complicated by wide variation in how unhealthy use is defined and which screening tools are used (Dagne et al. 2024). The Alcohol Use Disorders Identification Test Consumption (AUDIT‐C) (Bradley et al. 2007; Bush et al. 1998) is a widely used standardized screening questionnaire that is recommended by the United States Preventive Services Task Force (US Preventive Services Task Force et al. 2018) for screening for primary prevention. The AUDIT‐C assesses drinking frequency, drinking quantity, and binge level drinking, with cutoff scores that indicate unhealthy drinking (Bradley et al. 2007). The US National Institute on Alcohol Abuse and Alcoholism (NIAAA) guidelines are also widely used in practice, with recommended weekly limits as well as avoidance of binge drinking (National Institute on Alcohol Abuse and Alcoholism 2025). Establishing a PEth threshold for unhealthy drinking may further be complicated by inter‐individual variability in PEth formation and elimination (Helander et al. 2019), which may be influenced by clinical, demographic, or other characteristics (Skråstad et al. 2025; Torp et al. 2025). Although to date, large studies that have estimated cutoffs for unhealthy alcohol use have lacked population heterogeneity (Skråstad et al. 2023), thus no personalized cutoffs have been proposed.

To address this gap, we leveraged a previously aggregated set of studies that comprised over 10,000 individuals from the United States, multiple African countries, Russia, and Cambodia, and used two self‐reported measures of unhealthy use (AUDIT‐C and NIAAA) to identify a PEth cutoff to discriminate unhealthy alcohol use from low/no drinking. We also explored several subgroups of this sample, defined by clinical and demographic characteristics, to assess whether cutoffs that optimize sensitivity and specificity may differ for some groups.

## 2 Materials and Methods

### 2.1 Data Sources

Data are from 22 studies, including 21 previously aggregated in 2019 for an individual participant data meta‐analysis (Hahn et al. 2021), plus one additional cohort that recently tested for PEth levels, the Multicenter AIDS Cohort Study/Women's Interagency HIV Study Combined Cohort Study (MWCCS; D'Souza et al. 2021). Eleven studies were conducted solely among persons living with HIV (Table S1). All studies measured PEth 16:0/18:1 homologue concentrations with a lower limit of quantification < 8 ng/mL or lower and collected self‐reported current alcohol use with AUDIT‐C (Bradley et al. 2007; Bush et al. 1998) or another method from which AUDIT‐C could be calculated (e.g., Timeline Follow Back [TLFB]; Sobell and Sobell 1992); repeated measures were included when available. Exclusion criteria for the previous meta‐analysis were studies with fewer than 30 observations for which AUDIT‐C scores were ≥ 3 for women or ≥ 4 for men and studies of prisoners, persons in alcohol treatment, or clinical trials in which eligibility depended on self‐reported alcohol use without biomarker confirmation, as these groups may have reasons for misreporting alcohol consumption.

### 2.2 Study Measures

#### 2.2.1 Outcome

We considered two definitions of self‐reported unhealthy alcohol consumption: (1) the AUDIT‐C threshold for screening for “misuse” (Bradley et al. 2007): ≥ 3 for women or ≥ 4 for men, which has been shown to detect a range of unhealthy alcohol use patterns (US Preventive Services Task Force et al. 2018); and (2) the NIAAA definition of “heavy” drinking, which is > 7 drinks/week or ≥ 4 in any 1 day for women, and > 14/week or ≥ 5 in any 1 day for men (National Institute on Alcohol Abuse and Alcoholism 2025). We note that terminology varies in these definitions (misuse, heavy) and in the literature (including hazardous, risky). We use the term unhealthy to indicate levels above most clinical and national guidelines, though we acknowledge the ongoing debate whether any level of alcohol use is safe (Alvarez‐Mon et al. 2026). The AUDIT‐C definition of unhealthy alcohol use is a more conservative threshold than the NIAAA definition, because a positive AUDIT‐C score of 3 may indicate 1–2 drinks on 2–3 days per week, and a score of 4 may indicate 1–2 drinks on ≥ 4 days per week. The definitions also differ in that AUDIT‐C is a standardized questionnaire that asks about “typical” drinking frequency and quantity and any binge drinking. Included studies used reference periods of past month, 3 months, one year, or did not specify a period for “typical” drinking. For two studies that did not collect AUDIT‐C concurrently with PEth, we calculated AUDIT‐C scores from TLFB (Murnane 2025). We calculated the NIAAA drinking threshold with raw TLFB daily drink counts when available (past 30 or past 14 days), from summaries of weekly and daily consumption, or we approximated it from the individual AUDIT‐C questions as previously described (Murnane 2025).

#### 2.2.2 Predictor

The primary independent variable was PEth blood concentration (16:0/18:1 homologue), measured in ng/mL. Nanograms per milliliter can be converted to μmol/L by dividing by 703, the molecular weight of PEth 16:0/18:1. Concentrations > 1000 ng/mL (3% of the sample) were set to 1000 because PEth is only considered linear up to 1000 (Jones et al. 2011), and those below the lower limit of quantification were set to 1.

#### 2.2.3 Covariates

Because we previously found PEth sensitivity to differ by demographic and clinical factors (Hahn et al. 2021), we explored whether optimal PEth cutoffs may also differ for subgroups. First, we considered subgroups defined by geographic region, race/ethnicity, age, and sex, all of which were measured in all 22 studies. Geographic region was defined by study location as the African continent (including Uganda, South Africa, and Tanzania), Russia, Cambodia, or the United States (US). We considered region in part because of our prior finding of higher PEth sensitivity in studies based in Africa (Hahn et al. 2021), and in part because we have observed significant underreporting of alcohol use in African settings, possibly due to anticipated stigma (Asiimwe et al. 2015; Bajunirwe et al. 2014; Muyindike et al. 2017; Parry et al. 2023; Regenauer et al. 2020). Race/ethnicity was only collected in the US and was categorized as African American, White, and Other (including persons who identify as Latinx/Hispanic, Asian/Pacific Islander, Native American, mixed‐race, or not specified). We categorized age as < 35, 35–49, 50–64, or ≥ 65 years, and sex as male or female at birth given limited data on gender.

We also considered subgroups defined by clinical characteristics in the subsets of studies that collected these data. Body mass index (BMI) was categorized as overweight or obese (BMI ≥ 25 kg/m^2^) versus normal or underweight (< 25 kg/m^2^). HIV status was categorized as living with HIV and virologically suppressed, living with HIV and unsuppressed (including treatment naïve persons and those missing viral load data), or HIV negative. Anemia was classified as hemoglobin levels < 12 g/dL for females and < 13 g/dL for males. Liver fibrosis risk was defined by the FIB‐4 index, which is strongly associated with biopsy‐confirmed liver fibrosis, and calculated as follows: (age [years] * aspartate transaminase (AST) [U/L]) / (platelet count [10^9^/L] * √alanine transaminase (ALT)[U/L]) (Sterling et al. 2006). High fibrosis risk was classified as FIB‐4 scores ≥ 2.67 (Shah et al. 2009).

### 2.3 Statistical Methods

We first split the data into training and validation sets, randomly selecting and setting aside 10% of participants from each study for validation. In the remaining 90%, we evaluated optimal PEth thresholds, as detailed below, and assessed the sensitivity, specificity, and predictive values in the validation data. We chose to stratify our random splits by study, rather than leave out one or more studies as a validation dataset, because each study systematically differed by a variety of clinical, demographic, and regional factors, and we aimed to validate our results in a broadly generalizable cohort.

Within the training set, we used receiver operating characteristic (ROC) analysis to estimate and depict area under the ROC (AUROC) curves reflecting PEth's discrimination between no/low‐level alcohol use versus unhealthy use, separately for AUDIT‐C and NIAAA definitions. We first compared ROC curves by region using STATA's roccomp procedure (Cleves 2002) and estimated cutoffs with Youden's *J* (sensitivity + specificity − 1) (Youden 1950) to assess whether to proceed with pooled subgroup analyses. Because we observed large differences, we conducted all other subgroup analyses separately for studies based in the US and studies based in Africa. Due to small sample sizes for Russia and Cambodia, we did not conduct subgroup analyses within them. ROC curves by region are shown in Figure S1.

We then estimated AUROCs by subgroups defined by the covariates described above and estimated PEth cutoffs at Youden's *J*, which optimizes the avoidance of false positive and false negative results equally, using 1000 bootstrap samples with replacement. Bootstrap sampling was clustered by participant ID (to ensure all observations from the same individual were sampled together) and stratified by unhealthy alcohol use (yes/no) as per the reference standard definition. We report the median over the bootstrap samples as the “optimal” cutoff. Finally, we estimated the sensitivity, specificity, and positive and negative predictive values (PPV and NPV) of each cutoff in the validation dataset for subgroups with ≥ 50 observations. To allow comparison of PPV and NPV at the same prevalence across subgroups, we used the prevalence of unhealthy consumption per AUDIT‐C or NIAAA in the full sample for these estimates. All confidence intervals (AUROC, cutoffs, sensitivity/specificity, and predictive values) were estimated via the bootstrap percentile method. Considering that in some settings minimizing false positives is a priority, while in other settings reaching as many positives as possible is a priority, we then repeated these steps examining cutoffs to achieve ≥ 90% specificity or ≥ 90% sensitivity.

## 3 Results

Among 15,164 observations from 11,088 participants in 22 studies (Table S1), 1070 (7%) were missing either PEth concentrations or self‐reported alcohol use and were excluded from this analysis. After setting aside 10% (1023) for validation, the training dataset comprised 9161 participants with a median age of 48 years (interquartile range [IQR]: 34–57); 60% were male and 25% were from studies based in Africa (Table 1). Most participants were living with HIV, with 48% of the sample living with HIV and virologically suppressed and 25% with unsuppressed HIV. The distributions of demographic and clinical characteristics were comparable in the validation dataset (Table 1).

**TABLE 1:** Participant characteristics in training and test datasets.

	Training set	Test set
	*N* = 9161	*N* = 1023
**Demographic and clinical characteristics**, *n* (%)		
Geographic region		
US	6181 (67%)	692 (68%)
Africa	2261 (25%)	251 (25%)
Russia	539 (6%)	60 (6%)
Asia	180 (2%)	20 (2%)
Age in years, categorized		
< 35	2302 (25%)	233 (23%)
35–49	2672 (29%)	341 (33%)
50–64	3355 (37%)	377 (37%)
65+	830 (9%)	72 (7%)
Sex		
Male	5500 (60%)	595 (58%)
Female	3660 (40%)	428 (42%)
Body mass index (kg/m^2^)		
Under/normal weight (< 25)	3663 (46%)	393 (44%)
Overweight/obese (≥ 25)	4368 (54%)	510 (56%)
Anemia^a^	1586 (21%)	178 (21%)
FIB‐4 ≥ 2.67	600 (9%)	69 (9%)
HIV and viral suppression status		
HIV Negative	2456 (28%)	263 (27%)
HIV+ suppressed	4204 (48%)	480 (49%)
HIV+ not suppressed/unknown	2166 (25%)	243 (25%)
**Self‐reported alcohol use level**, *n* (%)		
AUDIT‐C		
No/low	5231 (57%)	587 (57%)
Unhealthy	3930 (43%)	436 (43%)
NIAAA		
No/low	6259 (68%)	688 (67%)
Unhealthy	2902 (32%)	335 (33%)
**Phosphatidylethanol blood concentration**, median (IQR)		
PEth 16:0/18:1, ng/mL	13 (BLQ‐100)	11 (BLQ‐93)

*Note:* Not all studies collected body mass index, hemoglobin, biomarkers to calculate FIB‐4, or HIV status.

Abbreviations: BLQ, below the limit of quantification; FIB‐4, Fibrosis index score; IQR, interquartile range.

^a^ Anemia was defined as hemoglobin < 12/13 g/dL Female/Male.

**FIGURE 1:**
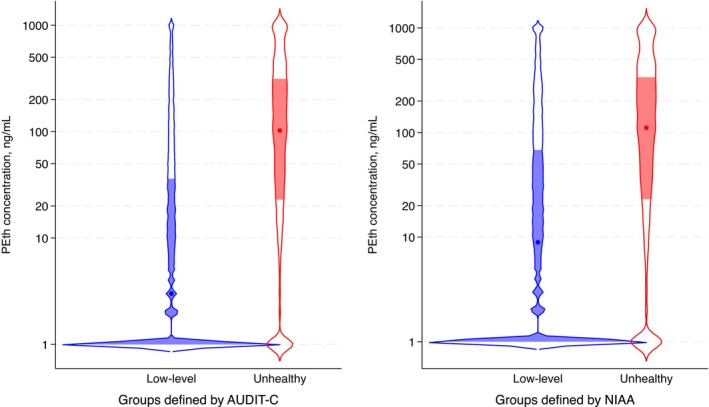
Distribution of PEth concentrations stratified by AUDIT‐C and NIAAA‐based thresholds for self‐reported unhealthy alcohol consumption. These violin plots show the interquartile range in the shaded regions and the median with a dot, while the outlines of the figures reflect the density of distribution of the data with varying width across PEth values.

Nearly half (43%) of participants self‐reported unhealthy alcohol use according to the AUDIT‐C definition while a third (32%) self‐reported consuming at unhealthy levels per the NIAAA definition (Table 1). The median PEth level was 13 ng/mL (IQR: below the lower limit of quantification [BLQ]‐100). Among those with self‐reported unhealthy alcohol use by the AUDIT‐C, the median PEth level was 102 ng/mL (IQR 23–313), while among those reporting no or low‐level consumption the median PEth was BLQ (IQR BLQ‐36 ng/mL; Figure 1). When classifying self‐reported unhealthy consumption or less by NIAAA thresholds, the distributions were similar (unhealthy median PEth 111 ng/mL, IQR 23–337; no/low‐level median PEth 9 ng/mL, IQR BLQ‐68).

**FIGURE 2:**
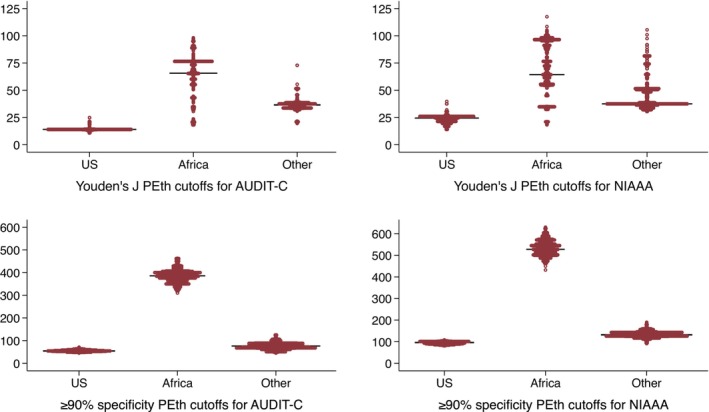
Distribution of 1000 bootstrapped estimates of PEth cutoffs by region and by definition of self‐reported unhealthy alcohol use, estimated with Youden's *J* or using ≥ 90% specificity. Red dots represent each estimate, black horizontal lines represent the median.

The AUROC reflecting PEth's ability to discriminate between self‐reported unhealthy drinking (per the AUDIT‐C) versus none/low‐level was 0.80 (95% CI: 0.79, 0.81) in studies from the US, 0.74 (95% CI: 0.72, 0.76) in studies from Africa, and 0.78 (95% CI: 0.75, 0.81) in studies from other regions (Table 2). The optimal PEth cutoff for self‐reported unhealthy drinking per AUDIT‐C estimated via Youden's *J* was 14.0 ng/mL (95% CI: 12.3, 18.6) in studies from the US, 65.7 ng/mL (95% CI: 19.3, 90.7) in studies from Africa, and 36.5 ng/mL (95% CI: 21.5, 50.6) in studies from other regions (Table 2, Figure 2). We note substantial variability of bootstrapped estimates in Africa as depicted in Figure 2 and reflected in the 95% confidence interval despite the large sample size. When using the AUDIT‐C to define self‐reported unhealthy drinking, most subgroup differences within the US were modest or had wide confidence intervals with substantial overlap, while within studies conducted in Africa they differed (with non‐overlapping confidence intervals) by sex and BMI. Sensitivity and specificity for Youden's *J* cutoffs were modest across subgroups with few reaching ≥ 80% for the AUDIT‐C definition of unhealthy drinking in the US and several below 60% in Africa.

**TABLE 2:** Using AUDIT‐C as the self‐reported unhealthy alcohol use reference standard: Area under receiver operating characteristic (AUROC), PEth cutoffs optimized at Youden's *J* (estimated via 1000 bootstrap samples in training data) and their sensitivity, specificity, positive and negative predictive values (estimated in test data), stratified by subgroups of interest.

Subgroup	*N* (training)	AUROC (95% CI)	Cutoff at Youden's *J* ng/mL	*N* (test)	Sensitivity (95% CI)	Specificity (95% CI)	PPV (95% CI)	NPV (95% CI)
**Region**								
United States	6433	0.80 (0.79, 0.81)	**14.0 (12.3, 18.6)**	729	73.0 (67.3, 79.1)	77.4 (73.2, 81.4)	70.9 (66.8, 74.9)	79.2 (75.5, 83.1)
African continent	4943	0.74 (0.72, 0.76)	**65.7 (19.3, 90.7)**	542	71.7 (64.4, 78.7)	65.2 (58.1, 72.7)	60.9 (56.3, 66.3)	75.4 (70.8, 79.9)
Other^a^	1304	0.78 (0.75, 0.81)	36.5 (21.5, 50.6)	143	57.8 (45.6, 69.8)	83.3 (71.9, 92.2)	72.4 (60.2, 84.1)	72.4 (66.3, 78.3)
**Subgroups within the United States**								
Race/ethnicity								
African American	3722	0.79 (0.77, 0.81)	22.0 (13.9, 26.7)	447	69.7 (62.1, 76.3)	76.6 (71.6, 81.3)	69.2 (64.1, 74.1)	77.0 (72.5, 81.4)
White	1663	0.83 (0.80, 0.85)	14.0 (12.9, 16.2)	170	67.1 (54.7, 78.7)	86.0 (78.4, 92.7)	78.3 (68.5, 87.7)	77.6 (71.4, 84.4)
Other	1042	0.81 (0.78, 0.83)	11.7 (9.6, 14.2)	111	67.4 (51.2, 81.8)	86.8 (78.5, 94.0)	79.4 (68.5, 89.9)	77.9 (70.0, 86.3)
Sex								
Female	2287	0.80 (0.78, 0.82)	14.1 (10.8, 25.4)	266	62.6 (53.0, 72.6)	79.0 (72.4, 84.5)	69.3 (62.2, 76.0)	73.7 (68.7, 79.5)
Male	4144	0.80 (0.79, 0.82)	13.9 (11.3, 20.9)	463	79.3 (73.0, 85.1)	76.8 (72.0, 81.6)	72.0 (67.9, 76.8)	83.1 (78.9, 87.4)
Age								
< 35	595	0.78 (0.75, 0.82)	10.5 (6.6, 22.8)	59	74.4 (58.2, 87.5)	70.0 (50.0, 88.9)	65.2 (51.2, 84.1)	78.3 (67.5, 88.4)
35–49	1737	0.77 (0.75, 0.80)	14.1 (10.3, 19.8)	227	73.3 (63.2, 82.0)	75.2 (68.0, 82.1)	69.0 (62.3, 76.0)	78.9 (72.7, 84.9)
50–64	3241	0.82 (0.80, 0.83)	23.2 (13.3, 26.3)	367	65.7 (57.0, 74.4)	82.0 (76.9, 87.1)	73.3 (67.6, 79.5)	76.0 (71.5, 80.9)
65+	860	0.86 (0.82, 0.89)	14.0 (11.3, 21.6)	76	66.7 (41.0, 94.6)	82.0 (72.6, 90.8)	73.6 (59.4, 86.6)	76.5 (64.5, 95.1)
BMI								
< 25 kg/m^2^	1881	0.81 (0.79, 0.83)	14.0 (11.2, 24.8)	203	81.2 (72.2, 89.7)	74.0 (66.1, 81.7)	70.2 (64.0, 77.3)	83.9 (77.6, 90.8)
≥ 25 kg/m^2^	3959	0.80 (0.79, 0.82)	14.1 (10.6, 16.9)	462	67.5 (59.7, 75.0)	79.3 (74.6, 83.5)	71.1 (66.1, 76.4)	76.4 (72.2, 81.0)
HIV								
HIV Negative	2231	0.83 (0.81, 0.84)	13.4 (10.0, 14.3)	240	73.2 (63.6, 81.2)	80.4 (73.9, 86.1)	73.8 (67.2, 80.3)	79.9 (74.2, 85.2)
HIV+ suppressed	3026	0.79 (0.77, 0.81)	16.5 (14.0, 24.4)	356	68.9 (59.8, 77.5)	78.1 (72.7, 83.3)	70.3 (64.5, 76.2)	76.9 (71.9, 82.1)
HIV+ viremia/unk	957	0.77 (0.73, 0.80)	23.6 (11.8, 43.5)	112	59.3 (43.6, 75.0)	77.6 (66.7, 87.5)	66.6 (54.3, 78.5)	71.6 (63.9, 80.2)
Anemia^b^								
Anemia	1289	0.79 (0.77, 0.82)	14.9 (8.0, 24.9)	146	68.1 (55.0, 81.2)	88.9 (82.4, 94.3)	82.2 (73.8, 90.3)	78.7 (72.2, 86.2)
No anemia	4362	0.82 (0.80, 0.83)	14.0 (11.0, 16.6)	497	72.7 (66.2, 79.4)	75.2 (70.0, 79.9)	68.8 (64.3, 73.5)	78.5 (74.5, 82.9)
Liver fibrosis risk^c^								
High	516	0.84 (0.81, 0.88)	28.7 (11.3, 30.0)	60	85.0 (68.3, 100.0)	77.5 (63.4, 89.3)	74.0 (62.6, 85.8)	87.3 (75.5, 100.0)
Low	5008	0.81 (0.79, 0.82)	14.2 (13.3, 19.4)	572	67.8 (60.9, 74.0)	78.6 (74.2, 82.7)	70.6 (66.1, 75.2)	76.4 (72.6, 80.1)
**Subgroups within the African continent**								
Sex								
Female	2123	0.79 (0.76, 0.81)	**18.8 (13.3, 23.7)**	248	79.0 (68.7, 88.8)	67.7 (57.8, 77.8)	64.8 (58.6, 72.5)	81.0 (74.3, 88.4)
Male	2820	0.70 (0.67, 0.72)	**88.8 (73.5, 156.5)**	294	73.7 (65.3, 81.8)	50.7 (39.7, 61.6)	53.0 (48.3, 58.9)	71.9 (65.6, 78.5)
Age								
< 35	2319	0.73 (0.70, 0.75)	19.0 (14.0, 77.1)	208	81.2 (71.7, 90.1)	66.1 (53.6, 78.0)	64.4 (57.5, 73.2)	82.4 (75.9, 89.3)
35–49	1988	0.77 (0.74, 0.80)	68.5 (22.5, 119.5)	257	76.2 (66.5, 84.6)	57.9 (46.9, 68.4)	57.7 (52.2, 64.3)	76.3 (69.4, 83.3)
50–64	585	0.76 (0.70, 0.81)	90.5 (56.0, 161.0)	73	70.6 (50.0, 88.3)	59.0 (38.5, 80.5)	56.5 (45.1, 72.5)	72.7 (59.4, 88.0)
65+	51	0.62 (0.39, 0.80)	82.0 (10.5, 389.0)	4				
BMI								
< 25 kg/m^2^	3684	0.76 (0.74, 0.78)	**76.5 (65.2, 97.2)**	397	75.8 (67.8, 83.3)	59.4 (51.3, 68.1)	58.5 (54.0, 63.6)	76.5 (71.0, 82.6)
≥ 25 kg/m^2^	1065	0.80 (0.76, 0.83)	**23.6 (14.7, 32.3)**	125	79.5 (61.5, 93.3)	67.9 (51.9, 83.8)	65.2 (54.8, 78.2)	81.5 (70.0, 93.1)
HIV								
HIV Negative	97	0.77 (0.68, 0.87)	16.5 (8.0, 168.0)	10				
HIV+ suppressed	1015	0.83 (0.80, 0.86)	40.0 (16.5, 95.5)	108	79.5 (70.3, 88.1)	60.0 (41.7, 78.1)	60.0 (50.2, 73.1)	79.5 (69.8, 87.5)
HIV+ viremia/unk	3649	0.75 (0.73, 0.77)	43.5 (18.3, 76.5)	404	82.5 (74.2, 90.0)	55.1 (47.2, 62.8)	58.1 (53.8, 62.9)	80.6 (73.7, 88.0)
Anemia^b^								
Anemia	544	0.75 (0.70, 0.80)	18.3 (13.5, 103.0)	60	80.0 (57.9, 100.0)	37.5 (17.6, 60.0)	49.1 (39.7, 59.4)	71.3 (50.8, 100.0)
No anemia	3531	0.76 (0.74, 0.78)	76.5 (32.8, 96.5)	391	71.7 (62.2, 80.2)	63.8 (55.1, 71.7)	59.9 (54.5, 65.7)	74.9 (69.3, 80.7)
Liver fibrosis risk^c^								
High	156	0.73 (0.63, 0.82)	65.5 (41.0, 429.5)	10				
Low	2526	0.74 (0.72, 0.76)	90.5 (59.9, 140.8)	284				

*Note: N* represent observations not individual participants. Bold is used to highlight cutoffs with non‐overlapping 95% confidence intervals.

Abbreviations: AUROC, area under receiver operating curve; BMI, body mass index; CI, confidence interval; NPV, negative predictive value; PPV, positive predictive value, unk, unknown.

^a^ Other region includes Russia and Cambodia.

^b^ Anemia is defined as hemoglobin < 12/13 g/dL Female/Male.

^c^ High liver fibrosis risk is defined as FIB‐4 scores ≥ 2.67.

Using the NIAAA threshold for self‐reported unhealthy drinking (Table 3), the optimal Youden's *J* PEth cutoff was 24.5 ng/mL in the United States (95% CI: 16.8, 27.4), 64.3 ng/mL in Africa (95% CI: 32.5, 100.2), and 37.5 ng/mL elsewhere (95% CI: 32.1, 81.5). As with AUDIT‐C, differences between other subgroups with non‐overlapping confidence intervals in Youden's *J* cutoffs were only observed in Africa by sex and BMI.

**TABLE 3:** Using NIAAA as the self‐reported unhealthy alcohol use reference standard: Area under receiver operating characteristic (AUROC), PEth cutoffs at Youden's *J* (estimated via 1000 bootstrap samples in training data) and their sensitivity, specificity, positive and negative predictive values (estimated in test data), stratified by subgroups of interest.

Subgroup	*N* (training)	AUROC (95% CI)	Cutoff at Youden's *J* ng/mL	*N* (test)	Sensitivity (95% CI)	Specificity (95% CI)	PPV (95% CI)	NPV (95% CI)
**Region**								
United States	6433	0.76 (0.75, 0.78)	**24.5 (16.8, 27.4)**	729	69.5 (63.1, 75.8)	80.2 (76.8, 83.5)	62.3 (57.8, 67.0)	84.8 (82.1, 87.6)
African continent	4943	0.69 (0.67, 0.71)	**64.3 (32.5, 100.2)**	542	76.9 (69.7, 83.6)	58.1 (51.6, 65.1)	46.4 (42.3, 51.0)	84.3 (80.4, 88.2)
Other^a^	1304	0.77 (0.74, 0.80)	37.5 (32.1, 81.5)	143	62.5 (47.6, 76.3)	78.5 (68.2, 87.4)	57.7 (45.9, 70.1)	81.6 (75.6, 87.7)
**Subgroups within the United States**								
Race/ethnicity								
African American	3722	0.75 (0.73, 0.77)	26.0 (21.9, 37.5)	447	68.8 (60.6, 76.1)	75.4 (70.4, 80.2)	56.8 (51.1, 62.6)	83.7 (80.2, 87.2)
White	1663	0.78 (0.75, 0.81)	20.9 (14.0, 30.4)	170	72.0 (58.4, 84.1)	85.0 (77.8, 91.2)	69.3 (58.5, 79.0)	86.6 (81.1, 92.0)
Other	1042	0.78 (0.75, 0.82)	15.0 (9.9, 22.5)	111	75.0 (57.4, 88.9)	86.1 (77.4, 93.6)	71.7 (59.9, 84.5)	88.0 (81.2, 94.3)
Sex								
Female	2287	0.78 (0.76, 0.80)	26.0 (14.2, 42.4)	266	58.5 (46.8, 70.6)	80.1 (74.7, 85.3)	58.0 (50.0, 67.0)	80.4 (76.1, 85.2)
Male	4144	0.76 (0.74, 0.77)	21.8 (15.4, 27.5)	463	74.2 (66.9, 81.4)	79.2 (74.6, 83.7)	62.7 (57.0, 68.6)	86.7 (83.3, 90.1)
Age								
< 35	595	0.71 (0.67, 0.76)	22.9 (6.8, 46.9)	59	70.4 (50.0, 86.4)	68.8 (51.9, 84.5)	51.4 (38.3, 69.3)	83.1 (73.6, 91.8)
35–49	1737	0.75 (0.73, 0.78)	15.0 (13.9, 30.9)	227	70.9 (60.2, 81.2)	70.9 (63.6, 78.1)	53.4 (46.4, 61.1)	83.8 (79.0, 88.9)
50–64	3241	0.77 (0.75, 0.79)	26.0 (18.9, 27.5)	367	72.4 (62.8, 81.2)	82.1 (77.3, 86.8)	65.5 (58.9, 72.7)	86.3 (82.5, 90.4)
65+	860	0.82 (0.77, 0.86)	32.2 (15.6, 50.8)	76	55.6 (20.0, 88.9)	91.0 (83.3, 97.0)	74.5 (48.7, 90.1)	81.3 (70.8, 94.7)
BMI								
< 25 kg/m^2^	1881	0.77 (0.74, 0.79)	24.0 (20.8, 44.5)	203	74.2 (62.1, 85.2)	75.2 (67.8, 82.4)	58.4 (51.5, 66.7)	86.1 (80.7, 91.5)
≥ 25 kg/m^2^	3959	0.76 (0.74, 0.78)	15.4 (14.0, 32.0)	462	74.0 (65.5, 81.6)	77.9 (73.3, 82.6)	61.2 (55.4, 67.1)	86.4 (82.7, 90.0)
HIV								
HIV Negative	2231	0.80 (0.78, 0.82)	20.4 (14.2, 32.1)	240	79.5 (68.7, 88.4)	83.2 (77.2, 88.8)	69.0 (61.3, 77.0)	89.6 (84.9, 93.9)
HIV+ suppressed	3026	0.74 (0.72, 0.76)	24.5 (14.2, 27.7)	356	65.6 (55.7, 74.9)	78.8 (73.3, 83.7)	59.3 (52.2, 66.5)	83.0 (78.8, 87.0)
HIV+ viremia/unk	957	0.75 (0.71, 0.79)	25.8 (13.2, 41.8)	112	62.2 (44.9, 77.3)	77.6 (66.2, 87.1)	56.7 (44.2, 70.3)	81.4 (74.5, 88.2)
Anemia^b^								
Anemia	1289	0.76 (0.73, 0.79)	24.9 (13.8, 64.0)	146	51.1 (37.1, 66.4)	89.1 (82.7, 95.0)	68.8 (56.4, 83.5)	79.5 (74.9, 85.0)
No anemia	4362	0.77 (0.75, 0.79)	24.5 (14.2, 29.0)	497	75.4 (68.0, 82.9)	77.7 (73.4, 81.9)	61.4 (56.6, 67.0)	87.0 (83.9, 90.5)
Liver fibrosis risk^c^								
High	516	0.82 (0.78, 0.86)	25.8 (14.6, 36.8)	60	85.0 (67.3, 100.0)	77.5 (63.6, 89.7)	64.0 (51.7, 79.1)	91.7 (83.2, 100.0)
Low	5008	0.76 (0.74, 0.78)	24.5 (14.0, 26.7)	572	64.3 (56.8, 72.4)	80.5 (76.7, 84.4)	60.8 (55.5, 66.7)	82.7 (79.7, 86.2)
**Subgroups within the African continent**								
Sex								
Female	2123	0.76 (0.72, 0.78)	**20.8 (18.3, 32.5)**	248	84.4 (72.8, 94.3)	61.6 (52.2, 71.4)	50.8 (45.2, 58.1)	89.4 (82.8, 95.5)
Male	2820	0.64 (0.61, 0.67)	**142.5 (81.1, 265.6)**	294	68.4 (57.7, 78.2)	53.6 (42.9, 63.4)	40.9 (35.7, 47.1)	78.3 (72.3, 84.2)
Age								
< 35	2319	0.68 (0.65, 0.71)	21.0 (18.3, 91.2)	208	88.1 (78.7, 95.6)	60.3 (48.5, 72.4)	51.1 (44.5, 59.7)	91.5 (85.6, 96.7)
35–49	1988	0.72 (0.69, 0.75)	69.8 (32.6, 136.5)	257	80.6 (69.5, 90.6)	51.8 (42.2, 61.4)	44.0 (38.9, 49.9)	85.0 (78.3, 92.0)
50–64	585	0.77 (0.71, 0.81)	153.2 (57.5, 317.5)	73	76.9 (40.0, 100.0)	58.3 (39.4, 76.8)	46.5 (31.7, 60.9)	84.3 (67.8, 100.0)
65+	51	0.69 (0.46, 0.87)	82.0 (10.5, 389.0)	4				
BMI								
< 25 kg/m^2^	3684	0.73 (0.70, 0.75)	**88.9 (61.5, 145.2)**	397	85.0 (77.3, 91.6)	57.2 (48.8, 65.3)	48.3 (44.0, 53.5)	89.1 (84.2, 93.6)
≥ 25 kg/m^2^	1065	0.75 (0.70, 0.79)	**32.3 (23.3, 36.0)**	125	76.2 (50.0, 93.8)	59.6 (46.5, 74.8)	47.0 (35.5, 59.7)	84.2 (71.4, 95.1)
HIV								
HIV Negative	97	0.92 (0.85, 0.97)	105.0 (11.5, 223.2)	10				
HIV+ suppressed	1015	0.71 (0.68, 0.74)	59.5 (27.4, 103.5)	108	75.0 (62.5, 86.9)	55.0 (43.0, 66.7)	44.0 (36.3, 52.4)	82.4 (73.7, 90.2)
HIV+ viremia/unk	3649	0.72 (0.69, 0.74)	45.6 (18.2, 98.4)	404	94.6 (88.5, 98.9)	51.2 (43.7, 59.0)	47.7 (44.0, 52.0)	95.3 (90.6, 99.0)
Anemia^b^								
Anemia	544	0.75 (0.70, 0.81)	63.8 (6.5, 107.5)	60	81.8 (57.1, 100.0)	53.1 (35.0, 72.8)	45.1 (35.1, 58.0)	86.1 (71.3, 100.0)
No anemia	3531	0.71 (0.68, 0.73)	86.4 (34.9, 105.4)	391	81.4 (72.2, 89.4)	59.2 (51.2, 67.5)	48.4 (43.5, 54.1)	87.1 (81.9, 92.2)
Liver fibrosis risk^c^								
High	156	0.62 (0.48, 0.73)	186.5 (14.5, 751.0)	10				
Low	2526	0.71 (0.68, 0.73)	96.5 (64.5, 145.5)	284				

*Note: N* represent observations not individual participants. Bold is used to highlight cutoffs with non‐overlapping 95% confidence intervals.

Abbreviations: AUROC, area under receiver operating curve; BMI, body mass index; CI, confidence interval; NPV, negative predictive value; PPV, positive predictive value, unk, unknown.

^a^ Other region includes Russia and Cambodia.

^b^ Anemia is defined as hemoglobin < 12/13 g/dL Female/Male.

^c^ High liver fibrosis risk is defined as FIB‐4 scores ≥ 2.67.

Cutoffs estimated to achieve least 90% specificity for unhealthy drinking per the AUDIT‐C were consistently higher than when using Youden's *J* (Tables 4 and 5), with the highest increases for studies conducted in Africa. PEth cutoffs for ≥ 90% specificity were 54.5 ng/mL (95% CI: 48.3, 62.9) in the US, 386.0 ng/mL (95% CI: 336.0, 441.5) in Africa, and 76.0 ng/mL (95% CI: 51.0, 106.0) elsewhere. Subgroup differences in both the US and Africa were more pronounced when prioritizing specificity, compared to using the Youden's *J*. For example, the cutoff for ≥ 90% specificity for unhealthy alcohol use by AUDIT‐C was 81.0 ng/mL (95% CI: 68.2, 98.0) for African American participants and 28.4 ng/mL (95% CI: 21.3, 35.3) for white participants in the US; in Africa, the cutoff was 465.0 ng/mL (95% CI: 399.0, 526.0) for participants with low BMI and 165.0 ng/mL (95% CI: 114.0, 216.0) for those with BMI ≥ 25. Similar patterns were seen using the NIAAA definition for self‐reported unhealthy alcohol use. Cutoffs estimated to achieve least 90% sensitivity for self‐reported unhealthy use per the AUDIT‐C and per NIAAA were nearly all below the limit of quantification.

**TABLE 4:** Using AUDIT‐C as the self‐reported unhealthy alcohol use reference standard: PEth cutoffs at 90% specificity (estimated via 1000 bootstrap samples in training data) and their sensitivity, positive and negative predictive values (estimated in test data), stratified by subgroups of interest.

Subgroups	*N* (training)	Cutoff at 90% specificity ng/mL	*N* (test)	Sensitivity (95% CI)	PPV (95% CI)	NPV (95% CI)
**Region**						
United States	6433	**54.4 (48.3, 62.9)**	729	51.8 (45.3, 58.0)	80.4 (75.5, 85.6)	71.3 (68.6, 74.1)
African continent	4943	**386.0 (336.0, 441.0)**	542	35.0 (27.0, 43.6)	63.7 (55.1, 74.5)	63.4 (60.8, 66.7)
Other^a^	1304	76.0 (51.0, 106.0)	143	41.0 (29.2, 52.9)	78.8 (64.0, 94.8)	67.3 (62.9, 72.4)
**Subgroups within the United States**						
Race/ethnicity						
African American	3722	**81.0 (68.2, 98.0)**	447	49.7 (41.4, 58.1)	78.5 (72.0, 84.6)	70.3 (66.9, 74.0)
White	1663	**28.4 (21.3, 35.3)**	170	57.1 (44.1, 69.3)	87.8 (79.3, 96.2)	74.4 (69.0, 80.3)
Other	1042	41.2 (32.7, 57.0)	111	46.5 (30.7, 63.5)	88.8 (76.2, 100.0)	70.3 (64.5, 77.6)
Sex						
Female	2287	56.1 (47.2, 73.0)	266	43.4 (33.8, 52.8)	77.4 (67.2, 86.1)	67.9 (63.9, 71.9)
Male	4144	53.4 (45.8, 64.0)	463	55.9 (47.9, 63.7)	81.6 (75.7, 87.8)	73.1 (69.5, 76.8)
Age						
< 35	595	39.0 (25.0, 60.0)	59	53.8 (37.5, 69.3)	80.2 (60.7, 100.0)	72.1 (64.6, 79.6)
35–49	1737	57.1 (45.0, 73.0)	227	48.9 (38.6, 59.8)	79.5 (69.7, 88.7)	70.1 (65.7, 75.0)
50–64	3241	69.0 (53.5, 82.0)	367	50.7 (41.5, 59.8)	81.7 (75.0, 88.7)	71.1 (67.5, 75.3)
65+	860	39.3 (30.4, 48.9)	76	40.0 (16.7, 66.7)	82.1 (60.1, 96.4)	67.4 (59.5, 78.9)
BMI						
< 25 kg/m^2^	1881	77.9 (57.1, 99.2)	203	53.8 (41.7, 65.8)	80.6 (71.7, 90.8)	72.1 (67.3, 77.8)
≥ 25 kg/m^2^	3959	50.0 (44.0, 58.1)	462	49.1 (41.0, 57.5)	82.8 (76.5, 89.2)	70.6 (67.4, 74.1)
HIV						
HIV Negative	2231	56.0 (47.9, 73.4)	240	51.5 (41.6, 61.7)	88.8 (81.2, 96.4)	72.2 (68.2, 76.6)
HIV+ suppressed	3026	53.0 (44.5, 64.1)	356	49.6 (40.5, 58.6)	76.0 (68.4, 83.6)	69.9 (66.0, 74.2)
HIV+ viremia/unk	957	57.0 (39.0, 97.0)	112	50.0 (33.1, 65.0)	75.8 (62.0, 89.3)	70.0 (63.6, 77.2)
Anemia^b^						
Anemia	1289	42.8 (34.0, 54.5)	146	48.9 (34.7, 62.8)	88.0 (78.5, 97.3)	71.1 (65.7, 77.3)
No anemia	4362	58.1 (49.0, 70.5)	497	52.5 (45.6, 59.6)	77.5 (71.8, 83.3)	71.2 (68.3, 74.4)
Liver fibrosis risk^c^						
High	516	120.0 (53.0, 177.0)	60	70.0 (50.0, 89.5)	80.9 (67.1, 95.4)	79.5 (69.3, 91.5)
Low	5008	53.7 (47.9, 63.0)	572	48.0 (40.7, 55.0)	78.8 (72.6, 84.6)	69.7 (66.7, 72.7)
**Subgroups within the African continent**						
Sex						
Female	2123	**162.0 (117.0, 194.2)**	248	32.1 (17.2, 47.2)	62.8 (42.9, 80.6)	62.6 (57.0, 68.4)
Male	2820	**575.0 (495.0, 651.0)**	294	28.2 (18.1, 37.3)	63.3 (49.5, 78.4)	61.8 (58.5, 64.9)
Age						
< 35	2319	266.0 (219.0, 329.0)	208	33.3 (20.5, 46.2)	77.9 (64.8, 91.2)	64.9 (60.9, 69.5)
35–49	1988	498.0 (417.5, 610.0)	257	31.4 (20.1, 43.4)	65.5 (49.2, 81.9)	62.8 (58.7, 67.4)
50–64	585	428.0 (329.0, 601.0)	73	52.9 (30.9, 75.0)	58.6 (43.3, 76.6)	66.9 (57.3, 79.2)
65+	51	336.0 (136.5, 869.0)	4			
BMI						
< 25 kg/m^2^	3684	**465.0 (399.0, 526.0)**	397	34.8 (25.8, 44.6)	65.7 (55.2, 76.2)	63.7 (60.5, 67.1)
≥ 25 kg/m^2^	1065	**165.0 (114.0, 216.0)**	125	29.5 (11.1, 48.8)	60.1 (32.9, 84.7)	61.6 (54.2, 69.0)
HIV						
HIV Negative	97	303.0 (70.0, 1000.0)	10			
HIV+ suppressed	1015	273.5 (195.0, 387.0)	108	48.7 (36.5, 60.0)	73.4 (56.0, 92.8)	69.1 (62.8, 75.1)
HIV+ viremia/unk	3649	405.0 (351.0, 473.5)	404	32.1 (22.0, 42.2)	60.6 (48.6, 72.5)	62.2 (58.6, 66.2)
Anemia^b^						
Anemia	544	339.0 (232.0, 459.5)	60	35.0 (14.3, 57.1)	44.8 (27.1, 65.6)	57.9 (48.6, 67.4)
No anemia	3531	454.0 (377.0, 502.0)	391	34.8 (25.4, 44.5)	70.3 (59.1, 81.5)	64.4 (61.1, 68.0)
Liver fibrosis risk^c^						
High	156	730.0 (498.0, 1000.0)	10			
Low	2526	490.0 (401.5, 581.5)	284			

*Note: N* represent observations not individual participants. Bold is used to highlight cutoffs with non‐overlapping 95% confidence intervals.

Abbreviations: AUROC, area under receiver operating curve; BMI, body mass index; CI, confidence interval; NPV, negative predictive value; PPV, positive predictive value, unk, unknown.

^a^ Other region includes Russia and Cambodia.

^b^ Anemia is defined as hemoglobin < 12/13 g/dL Female/Male.

^c^ High liver fibrosis risk is defined as FIB‐4 scores ≥ 2.67.

**TABLE 5:** Using NIAAA as the self‐reported unhealthy alcohol use reference standard: PEth cutoffs at 90% specificity (estimated via 1000 bootstrap samples in training data) and their sensitivity, positive and negative predictive values (estimated in test data), stratified by subgroups of interest.

Subgroup	*N* (training)	Cutoff at 90% specificity ng/mL	*N* (test)	Sensitivity (95% CI)	PPV (95% CI)	NPV (95% CI)
**Region**						
United States	6433	**96.3 (84.1, 103.4)**	729	47.3 (40.1, 54.3)	74.9 (68.5, 81.2)	78.9 (76.5, 81.1)
African continent	4943	**528.0 (475.0, 600.0)**	542	31.5 (21.3, 41.6)	52.7 (41.8, 64.2)	72.9 (70.0, 76.0)
Other^a^	1304	**132.0 (112.0, 159.0)**	143	35.9 (21.3, 49.2)	69.0 (49.7, 86.6)	75.4 (71.1, 79.3)
**Subgroups within the United States**						
Race/ethnicity						
African American	3722	**117.2 (103.9, 140.3)**	447	47.1 (38.3, 56.8)	71.0 (62.5, 79.6)	78.5 (75.6, 81.7)
White	1663	**71.0 (56.1, 85.8)**	170	48.0 (34.7, 61.8)	84.4 (72.6, 96.3)	79.7 (75.6, 84.2)
Other	1042	61.0 (51.0, 78.0)	111	37.5 (19.4, 56.3)	77.7 (56.2, 94.8)	76.3 (71.3, 82.1)
Sex						
Female	2287	84.0 (72.7, 103.9)	266	46.2 (33.5, 59.1)	75.7 (63.4, 86.0)	78.6 (74.8, 82.7)
Male	4144	99.9 (87.7, 107.0)	463	47.7 (39.2, 56.6)	74.2 (65.7, 83.5)	78.9 (76.3, 81.9)
Age						
< 35	595	87.0 (66.6, 115.5)	59	33.3 (14.3, 52.0)	55.7 (30.3, 93.9)	73.6 (67.6, 80.1)
35–49	1737	87.0 (63.3, 102.5)	227	39.2 (28.4, 50.6)	66.1 (54.4, 79.0)	76.0 (72.7, 79.7)
50–64	3241	104.0 (93.6, 119.1)	367	59.0 (47.5, 69.0)	80.2 (71.6, 87.4)	82.9 (79.0, 86.5)
65+	860	81.0 (58.1, 106.0)	76	44.4 (12.5, 77.8)	77.8 (42.9, 96.0)	78.2 (69.2, 90.3)
BMI						
< 25 kg/m^2^	1881	**118.0 (104.0, 145.6)**	203	54.8 (41.4, 68.5)	72.2 (60.7, 84.2)	80.9 (76.5, 86.0)
≥ 25 kg/m^2^	3959	**81.9 (72.4, 97.4)**	462	45.7 (36.2, 54.2)	75.8 (67.1, 84.5)	78.5 (75.6, 81.2)
HIV						
HIV Negative	2231	101.0 (83.9, 114.1)	240	52.1 (39.9, 62.6)	87.2 (76.7, 95.8)	81.0 (77.4, 84.6)
HIV+ suppressed	3026	89.0 (74.5, 103.0)	356	43.8 (34.0, 53.7)	68.2 (58.1, 78.1)	77.3 (74.3, 80.6)
HIV+ viremia/unk	957	104.0 (64.0, 130.0)	112	46.7 (26.7, 62.2)	67.8 (50.4, 87.5)	78.1 (72.3, 83.6)
Anemia^b^						
Anemia	1289	**58.0 (47.5, 84.0)**	146	42.2 (27.2, 57.5)	74.1 (59.0, 88.5)	77.4 (73.0, 82.3)
No anemia	4362	**99.0 (87.2, 108.1)**	497	50.7 (42.3, 59.5)	75.6 (68.0, 82.8)	79.9 (77.3, 82.9)
Liver fibrosis risk^c^						
High	516	**176.0 (115.5, 268.0)**	60	70.0 (50.0, 88.9)	86.8 (70.5, 100.0)	87.1 (79.7, 94.8)
Low	5008	**89.0 (78.5, 100.0)**	572	45.9 (36.9, 54.5)	73.7 (66.6, 80.7)	78.4 (75.7, 81.1)
**Subgroups within the African continent**						
Sex						
Female	2123	**248.0 (205.0, 293.5)**	248	40.0 (21.9, 55.4)	58.6 (41.8, 75.2)	75.4 (70.3, 80.4)
Male	2820	**718.0 (642.0, 806.0)**	294	22.4 (12.4, 34.0)	46.3 (32.1, 59.6)	70.6 (68.0, 73.7)
Age						
< 35	2319	371.0 (318.0, 458.0)	208	29.9 (17.1, 43.3)	64.3 (47.1, 82.7)	73.6 (70.2, 77.3)
35–49	1988	697.0 (617.0, 785.0)	257	25.8 (12.9, 39.8)	50.7 (34.2, 68.4)	71.6 (68.3, 75.2)
50–64	585	501.0 (415.5, 651.0)	73	69.2 (28.6, 93.3)	56.6 (34.3, 75.2)	83.8 (68.9, 96.1)
65+	51	441.0 (138.0, 1000.0)	4			
BMI						
< 25 kg/m^2^	3684	**612.0 (545.0, 683.0)**	397	32.7 (22.2, 44.1)	55.4 (44.5, 66.5)	73.4 (70.6, 76.5)
≥ 25 kg/m^2^	1065	**253.0 (210.0, 318.0)**	125	19.0 (4.0, 39.0)	36.8 (8.5, 72.5)	69.0 (63.5, 75.1)
HIV						
HIV Negative	97	195.0 (69.0, 445.0)	10			
HIV+ suppressed	1015	679.0 (579.0, 799.5)	108	27.1 (14.0, 39.6)	52.2 (32.1, 76.0)	72.0 (68.0, 75.9)
HIV+ viremia/unk	3649	507.0 (460.0, 589.0)	404	33.8 (18.5, 49.3)	54.4 (39.1, 66.9)	73.6 (69.5, 78.3)
Anemia^b^						
Anemia	544	455.0 (347.0, 809.0)	60	36.4 (8.3, 66.7)	43.3 (15.8, 72.3)	72.1 (63.1, 83.0)
No anemia	3531	599.0 (532.0, 647.0)	391	31.4 (19.6, 44.3)	53.6 (41.0, 65.8)	73.0 (69.8, 76.6)
Liver fibrosis risk^c^						
High	156	934.0 (645.0, 1000.0)	10			
Low	2526	644.0 (583.5, 752.5)	284			

*Note: N* represent observations not individual participants. Bold is used to highlight cutoffs with non‐overlapping 95% confidence intervals.

Abbreviations: AUROC, area under receiver operating curve; BMI, body mass index; CI, confidence interval; NPV, negative predictive value; PPV, positive predictive value, unk, unknown.

^a^ Other region includes Russia and Cambodia.

^b^ Anemia is defined as hemoglobin < 12/13 g/dL Female/Male.

^c^ High liver fibrosis risk is defined as FIB‐4 scores ≥ 2.67.

## 4 Discussion

In this multinational and clinically diverse study sample of over 10,000 individuals, we found substantial differences in optimal PEth cutoffs for self‐reported unhealthy drinking by region and by some subgroups within regions. Using Youden's *J*, optimal cutoffs for unhealthy drinking by AUDIT‐C were 14.0, 65.7, and 36.5 ng/mL for the US, Africa, and other regions, respectively. NIAAA criteria to define unhealthy drinking yielded a modestly higher cutoff (24.5 ng/mL) than AUDIT‐C in the US, which mirrors the higher threshold for the level of alcohol consumption that is considered unhealthy using NIAAA, though the NIAAA cutoff in African studies (64.3 ng/mL) did not differ from that of AUDIT‐C. Our Youden's *J* cutoffs in studies from Africa are closely aligned with a recent population‐based study from Norway of nearly 25,000 adults that estimated a Youden's *J* cutoff at 61.2 to detect ≥ 2 drinks per day on average in the past 12 months (Skråstad et al. 2023); their estimated cutoff of 40.1 ng/mL for ≥ 1 drink per day falls between our regionally estimated cutoffs for unhealthy alcohol use. The AUROCs, sensitivity, and specificity across cutoffs in our data were modest, suggesting limited discrimination of self‐reported unhealthy drinking across levels of PEth. Our observed variability in cutoffs by region and some subgroups may be due to differences in reporting bias and in alcohol metabolism, which we discuss further below.

Underreported alcohol use could cause higher PEth cutoffs when greater amounts of alcohol are actually consumed than what is self‐reported. Underreporting can be attributable to several factors such as interviewer skill (McNeely et al. 2018; Williams et al. 2015), intentional underreporting due to internalized or anticipated stigma (Parry et al. 2023; Sudhinaraset et al. 2016; Zewdu et al. 2019) or other fears of negative consequences such as loss of driving privileges, or unintentional misreporting. In the African setting, where we estimated the highest cutoffs and observed the highest within‐group variability, we and others have previously observed high underreporting using PEth as a gold standard (Asiimwe et al. 2015; Bajunirwe et al. 2014; Muyindike et al. 2017; Parry et al. 2023). Persons living with HIV in South Africa have discussed intentional underreporting due to lived experiences of stigmatizing language coming from healthcare workers (Regenauer et al. 2020). Moreover, the quantity of ethanol in a single “drink” can vary widely and is unregulated in traditional brews in many African settings. Indeed, the definition of a “standard drink” varies internationally, typically ranging from 10 g of pure ethanol, as in many European countries, to 14 g, as in the United States (Kalinowski and Humphreys 2016). Consumers are commonly uninformed of these standards, which could bias self‐reported levels of use (Kerr and Stockwell 2012).

Variability in PEth formation and elimination may be influenced by a variety of clinical and individual physical characteristics including sex and age, body composition, hepatic function, hematologic factors such as hematocrit and red blood cell lifespan, and comorbid conditions that alter erythrocyte turnover (Cederbaum 2012; Torp et al. 2025). We did not observe meaningful differences in optimal cutoffs by age or sex in the US, though in studies from Africa women had substantially lower thresholds than men (e.g., 18.8 vs. 88.8 ng/mL for AUDIT‐C/Youden's *J*). While women reach a higher blood alcohol concentration at the same volume of alcohol consumption (Ulwelling and Smith 2018), both AUDIT‐C and NIAAA definitions of unhealthy use differ by sex and therefore may already account for this, which may explain the comparable cutoffs observed in the US. Indeed, Skråstad did find that at the same level of self‐reported intake, males had lower PEth levels than females (Skråstad et al. 2025). However, the reasons for the sex differences in Africa remain unclear. In a study of repeated PEth measures over time during alcohol detoxification, no difference was observed in the rate of PEth elimination by sex (Helander et al. 2019).

We found lower PEth cutoffs for persons with high BMI in studies from Africa across all cutoff definitions. This finding is consistent with prior observations of lower PEth sensitivity associated with high BMI (Hahn et al. 2021; Wang et al. 2018), that is, if lower PEth concentrations are reached at the same level of alcohol consumption this could also result in reduced PEth detection. In contrast, higher body fat has been associated with higher blood alcohol concentration (Ulwelling and Smith 2018), yet how body fat impacts PEth formation or elimination has not been examined. Notably, a population‐based study in Norway found overall weight as well as higher soft lean mass (body weight minus that of fat and bone) were both associated with lower PEth levels at the same amount of self‐reported alcohol consumption (Skråstad et al. 2025).

More subgroup differences emerged when we considered PEth cutoffs at ≥ 90% specificity. For both AUDIT‐C and NIAAA definitions of self‐reported unhealthy alcohol use, we found higher ≥ 90% specificity PEth cutoffs for African Americans compared to other groups. This is consistent with our prior finding of higher PEth sensitivity for detecting self‐reported unhealthy alcohol use in this group (Hahn et al. 2021; Jain et al. 2024). For the NIAAA definition of self‐reported unhealthy alcohol use, but not the AUDIT‐C definition, the ≥ 90% specificity PEth cutoffs in the United States were lower for those with high BMI (consistent with studies from Africa), lower for those with anemia, and higher for those with high liver fibrosis risk. While the lower cutoff for anemia and higher cutoff for fibrosis risk were not observed with non‐overlapping confidence intervals in Africa, these subgroups were small and the direction of estimates was consistent with the US.

A lower cutoff for anemia is supported by our prior finding of lower PEth sensitivity for persons with anemia (Hahn et al. 2021). Because PEth accumulates on red blood cells (Tawiah et al. 2022; Torp et al. 2025; Varga and Alling 2002), reduced red blood cell concentration in conditions such as anemia may result in lower whole blood PEth concentrations (Bartel et al. 2023; White et al. 2023). While lower hemoglobin levels are more prevalent among African Americans (Weyand and McGann 2021), this did not translate to lower PEth cutoffs in this subgroup in our study. A higher cutoff for high liver fibrosis risk is consistent with our finding of higher PEth sensitivity (Hahn et al. 2021). This finding may be explained, in part, by liver damage slowing alcohol elimination (Cederbaum 2012) thus allowing for greater PEth accumulation. Additionally, one study found that chronic heavy drinking is associated with higher rates of PEth formation at the same level of consumption as healthy controls (Varga and Alling 2002), which is relevant for persons with fibrosis given that heavy drinking is associated with liver damage. Still, one study among persons in a detoxification setting found opposing mechanisms influencing PEth elimination in relation to liver fibrosis (Bartel et al. 2023). At roughly the same level of alcohol intake, they found markedly elevated serum alcohol levels among persons with cirrhosis compared to those without. However, PEth elimination was only slightly slower, and no association was observed between PEth elimination and earlier stages of fibrosis. Instead, they found evidence of enhanced red blood cell turnover in association with cirrhosis and with faster PEth elimination, potentially counter‐balancing the impact of slower alcohol elimination.

We did not observe appreciable differences in cutoffs by HIV status. HIV infection is associated with a number of comorbidities, which may influence PEth metabolism (Torp et al. 2025). HIV can lead to an increased risk of red blood cell degradation (Marchionatti and Parisi 2021) but may also impact other cell types (Obeagu and Goryacheva 2025). Thus, red blood cell concentration in whole blood could vary in either direction, with an uncertain impact on PEth concentrations. In two controlled studies of blood alcohol concentration shortly after consumption, one found higher ethanol concentrations in persons with untreated HIV compared to those with treated HIV (McCance‐Katz et al. 2012), while another found lower concentrations among men living with HIV (most of whom were on treatment) than HIV negative men (Shuper et al. 2018).

We calculated cutoffs using Youden's *J*, which optimizes the sum of sensitivity and specificity and is comparable to most studies of PEth cutoffs. This balance may be most appropriate when minimizing false negatives and false positives carry equal weight, though optimizing sensitivity may be preferred for screening when the barrier to intervention is low. However, in legal and employment settings (Ulwelling and Smith 2018) or clinical settings when greater certainty that a positive result is truly positive is desired, optimizing specificity may be preferred. Therefore, future work will need to determine priorities for PEth cutoffs, or produce alternative cutoffs for clearly delineated purposes (e.g., legal).

This research is limited by the reliance on self‐report. While we excluded studies for which we felt underreporting was likely, inaccurate reporting is always a risk. With only a few small studies examining PEth with controlled and monitored alcohol consumption (Aboutara et al. 2023; Bartel et al. 2023; Stöth et al. 2023), our approach in using self‐report as the reference standard is strengthened by a clinically and demographically diverse sample of over 10,000 individuals, and is consistent with the majority of prior research aiming to identify cutoffs (Skråstad et al. 2023).

Another limitation is that our definitions of self‐reported unhealthy alcohol use do not distinguish between drinking patterns and can be reached either by binge drinking or by regular lower‐level intake. Furthermore, neither definition adequately characterizes recency, particularly for peak levels of consumption, which likely influences PEth concentrations (Ulwelling and Smith 2018) but deserves more study. Importantly, AUDIT‐C asks about typical drinking—over the past year, three months, or as short as past 30 days in our included studies—and these different reference periods may have contributed to the variability in our estimated cutoffs. For studies in which the NIAAA threshold was estimated with Timeline follow back, the 30 day reference period would be more closely aligned with the period reflected in PEth concentrations. Another limitation of our study is that our regional subgroup of studies from Africa includes a mix of low and moderate income countries with different cultural norms. While our concerns about the limitations of self‐report in our data preclude recommendation of clinical use of our estimated cutoffs, we remain optimistic that appropriate PEth cutoffs for unhealthy alcohol use are attainable. We recommend that emerging technologies such as machine learning and wearable biosensors that measure alcohol use in real‐time be leveraged to provide more accurate measurement of alcohol use, including patterns, recency, and quantity, with larger and more diverse study samples (i.e., including a broad range by BMI or body fat composition, race/ethnicity, liver disease, HIV status, and anemia) than is possible in alcohol administration studies.

In conclusion, we found substantial geographic variability of optimal PEth cutoffs, and some differences by clinical and demographic characteristics. We believe the high variability may be attributable to differences in PEth formation and elimination as well as differences in reporting bias between subgroups. Given the increasing use of PEth in clinical care, particularly in liver disease management (Dumortier et al. 2025; Tavaglione et al. 2025; Torp et al. 2025; Watt et al. 2025), research on the factors that influence PEth formation and elimination, using objective measurement such as biosensors, is needed to aid interpretation of PEth results. A threshold that adequately detects unhealthy drinking is needed to facilitate primary prevention of alcohol‐related morbidity and mortality.

## Funding

This work was supported by the National Institutes of Health, U01 AA026223, U01 HL146242, K24 AI108516, R01 DK109823, U01 AA020776, R01 AA018631, U01 AA026226, R01 AI119037, P30 AI027763, R01 DA016017, U01 AA022001, U10 AA013566, K24 AA022586, R01 AA018096, K23 AA024503, U01 AA020790, U01 AA020795, U01 AA020784, P60 AA009803, R21 AA024535, R01 AA017911, U01 AA020780, R01 AA022222, K01 AA021671, U01 AA020797, NIH R24AA019661, NIH R01 DA051464, NIH P01 AA029545, NIH U24 AA020794, NIH T32 DA017629, NIH R01 DA016065. Bill and Melinda Gates Foundation, OPP1056051.

## Conflicts of Interest

The authors declare no conflicts of interest.

## Supporting information


**Table S1:** Participant characteristics by study.
**Figure S1:** Area under receiving operator curves by region—depicting PEth discrimination of self‐reported unhealthy alcohol use via AUDIT‐C and NIAAA reference standards for unhealthy alcohol use.

## Data Availability

We obtained permission for use of these data from 22 studies for this analysis and did not obtain permission to share forward. We recommend interested parties contact the PIs of each dataset individually to request permission.
