# Supplementary material for: Exploring Phosphatidylethanol Cutoffs for Self‐Reported Unhealthy Alcohol Use: An International Multi‐Site Analysis
Source: Alcohol Clin Exp Res (Hoboken). 2026 Jun 25;50(6):e70361. doi: 10.1111/acer.70361 (PMC13296258; doi:10.1111/acer.70361)
Supplement: Supplementary file 1 — Table S1: Participant characteristics by study. Figure S1: Area under receiving operator curves by region—depicting PEth discrimination of self‐reported unhealthy alcohol use via AUDIT‐C and NIAAA reference standards for unhealthy alcohol use. [file ACER-50-0-s001.docx]

Supplementary materials

Supplementary Table 1. Participant characteristics by study.

|  |  |  |  |  | Body mass index, kg/m^2^ | | | |  |  | HIV Status | | |
| --- | --- | --- | --- | --- | --- | --- | --- | --- | --- | --- | --- | --- | --- |
|  |  | Age | Male | Female | <18.5 | 18.5-24.9 | 25-29.9 | ≥30 | Anemia | High FIB4 | Negative | +/supp | +/viremia |
| ADEPT | 429 | 32 (27-41) | 134 (31%) | 295 (69%) | 28 (7%) | 268 (62%) | 88 (21%) | 45 (10%) | 39 (9%) | 8 (6%) | 0 | 68 (16%) | 361 (84%) |
| ADEPTT | 301 | 40 (33-47) | 147 (49%) | 154 (51%) | 31 (10%) | 185 (61%) | 50 (17%) | 35 (12%) | 33 (11%) | 5 (2%) | 0 | 281 (93%) | 20 (7%) |
| Boston | 249 | 50 (44-56) | 157 (63%) | 92 (37%) | 10 (4%) | 94 (38%) | 83 (33%) | 62 (25%) | 79 (32%) | 40 (16%) | 0 | 190 (76%) | 59 (24%) |
| BREATH | 346 | 29 (24-37) | 189 (55%) | 157 (45%) | 41 (12%) | 233 (67%) | 56 (16%) | 16 (5%) | 56 (16%) |  | 0 | 71 (21%) | 275 (79%) |
| CHAMPS | 135 | 54 (50-59) | 80 (59%) | 55 (41%) | 7 (5%) | 54 (40%) | 41 (31%) | 32 (24%) | 31 (36%) | 22 (27%) | 0 | 115 (85%) | 20 (15%) |
| DIPT | 680 | 39 (32-47) | 470 (69%) | 210 (31%) | 137 (20%) | 439 (65%) | 83 (12%) | 21 (3%) | 80 (12%) | 48 (7%) | 0 | 638 (94%) | 42 (6%) |
| HOLIDAY | 104 | 67 (60-74) | 81 (79%) | 22 (21%) | 0 | 26 (27%) | 42 (44%) | 28 (29%) | - | - |  |  |  |
| INVOICE | 100 | 44 (32-50) | 100 (100%) | 0 |  |  |  |  |  |  | 0 | 47 (47%) | 53 (53%) |
| Liver Pts | 139 | 53 (46-58) | 75 (54%) | 64 (46%) |  |  |  |  | 56 (43%) | 70 (54%) | 67 (97%) | 0 | 2 (3%) |
| META | 428 | 32 (27-40) | 160 (37%) | 267 (63%) | 51 (12%) | 220 (53%) | 80 (19%) | 65 (16%) |  | - | 0 | 41 (10%) | 387 (90%) |
| MIAMI | 57 | 55 (53-58) | 37 (65%) | 20 (35%) | 2 (4%) | 19 (33%) | 18 (32%) | 18 (32%) |  | 4 (7%) | 24 (42%) | 5 (9%) | 28 (49%) |
| MWCCS | 3,400 | 56 (47-62) | 1,411 (42%) | 1,989 (58%) | 41 (1%) | 684 (22%) | 958 (30%) | 1,497 (47%) | 673 (21%) | 130 (4%) | 1,169 (34%) | 1,964 (58%) | 267 (8%) |
| NOAH | 365 | 50 (41-56) | 252 (69%) | 113 (31%) | 16 (4%) | 140 (39%) | 106 (29%) | 100 (28%) | 102 (29%) | 34 (10%) | 0 | 312 (85%) | 53 (15%) |
| Russia | 351 | 33 (30-37) | 248 (71%) | 103 (29%) | 19 (5%) | 266 (76%) | 54 (15%) | 12 (3%) | 26 (10%) | 41 (18%) | 0 | 41 (12%) | 310 (88%) |
| Russian Women | 250 | 30 (28-32) | 0 | 250 (100%) |  |  |  |  |  |  | 0 | 230 (92%) | 20 (8%) |
| Sihanouk | 200 | 24 (21-29) | 100 (50%) | 100 (50%) |  |  |  |  |  |  | 188 (94%) | 0 | 11 (6%) |
| Tanzania | 202 | 22 (21-24) | 161 (80%) | 41 (20%) |  |  |  |  |  |  |  |  |  |
| Trauma Patients | 251 | 53 (33-65) | 187 (75%) | 64 (25%) | 4 (2%) | 59 (31%) | 64 (34%) | 64 (34%) | 74 (30%) | 27 (30%) | 251 (100%) | 0 | 0 |
| TRUST | 156 | 37 (28-49) | 101 (65%) | 55 (35%) | 85 (54%) | 61 (39%) | 8 (5%) | 2 (1%) | 99 (63%) | 2 (1%) | 107 (69%) | 11 (7%) | 38 (24%) |
| UFO | 137 | 25 (22-27) | 101 (74%) | 36 (26%) |  |  |  |  |  |  | 125 (96%) | 0 | 5 (4%) |
| VACS | 2,656 | 52 (47-57) | 2,517 (95%) | 139 (5%) | 57 (2%) | 949 (36%) | 922 (35%) | 717 (27%) | 589 (22%) | 308 (12%) | 935 (35%) | 1,105 (42%) | 616 (23%) |
| Piano | 152 | 22 (20-24) | 61 (40%) | 91 (60%) | 1 (1%) | 119 (78%) | 31 (20%) | 1 (1%) | 11 (10%) | 0 | 152 (100%) | 0 | 0 |

Study data collection periods and citations: ADEPT 2012-2015 (Hahn et al., 2018); ADEPTT 2017-2019 (Muyindike et al., 2022); Boston 2012-2016 (Saitz et al., 2018); BREATH 2011-2014 (Hahn et al., 2016); CHAMPS 2015-2017 (Irvin et al., 2020); DIPT 2018-2022 (Lodi et al., 2021); HOLIDAY 2014-2019 (Marcus et al., 2021); INVOICE 2015-2019; Liver Pts 2009-2012 (Stewart et al., 2014); META 2015-2016 (Magidson et al., 2019); MIAMI 2018-2019 (Cook et al., 2024); MWCCS 2020-2023 (D’Souza et al., 2021); NOAH 2015-2017 (Ferguson et al., 2020); Russia 2012-2017 (So-Armah et al., 2019); Russian Women 2013-2014 (Littlefield et al., 2017); Sihanouk 2011 (Couture et al., 2016); Tanzania 2014 (Francis et al., 2015); Trauma Patients 2017-2019 (Afshar et al., 2022); TRUST 2017-2019 (Myers et al., 2018); UFO 2010-2011 (Jain et al., 2014); VACS 2005-2007 (Eyawo et al., 2018); Piano 2017-2018 (Piano et al., 2015)

Supplementary Figure 1. Area under receiving operator curves by region -- depicting PEth discrimination of self-reported unhealthy alcohol use via AUDIT-C and NIAAA reference standards for unhealthy alcohol use
